# High Muscle Expression of IGF2BP1 Gene Promotes Proliferation and Differentiation of Chicken Primary Myoblasts: Results of Transcriptome Analysis

**DOI:** 10.3390/ani14142024

**Published:** 2024-07-09

**Authors:** Jintang Luo, Zhuliang Yang, Xianchao Li, Cong Xiao, Hong Yuan, Xueqin Yang, Biyan Zhou, Yan Zheng, Jiayi Zhang, Xiurong Yang

**Affiliations:** 1 College of Animal Science and Technology, Guangxi University, Nanning 530004, China; ljt1903010101@163.com (J.L.); yangzl@st.gxu.edu.cn (Z.Y.); lixianchao2024@163.com (X.L.); xiaocong81875@163.com (C.X.); yuanhong19970610@163.com (H.Y.); yangxueqin1109@163.com (X.Y.); zhoubiyan07@163.com (B.Z.); shimoyi1@sina.com (Y.Z.); zhangjiay07@163.com (J.Z.); 2 Guangxi Key Laboratory of Animal Breeding, Disease Control and Prevention, Nanning 530004, China

**Keywords:** IGF2BP1, myoblasts, proliferation, differentiation, RNA-seq

## Abstract

**Simple Summary:**

Muscle development plays a pivotal role in the growth process of chickens. Clarifying the mechanisms of muscle development holds significant scientific and economic value. In this study, transcriptome sequencing was conducted on muscle tissues from two broilers with differing growth rates, identifying Insulin–like growth factor 2 mRNA binding protein 1 (IGF2BP1) as a potential participant in chicken muscle development via bioinformatics analysis. The identified genes were further validated using overexpression and knockdown approaches. It was found that IGF2BP1 is highly expressed in chicken muscle tissues, and the experimental results indicated that IGF2BP1 expression promotes the proliferation and differentiation of chicken primary myoblasts (CPMs). These findings offer new insights into the regulation of muscle development.

**Abstract:**

Muscle development is a multifaceted process influenced by numerous genes and regulatory networks. Currently, the regulatory network of chicken muscle development remains incompletely elucidated, and its molecular genetic mechanisms require further investigation. The Longsheng-Feng chicken, one of the elite local breeds in Guangxi, serves as an excellent resource for the selection and breeding of high-quality broiler chickens. In this study, we conducted transcriptome sequencing of the pectoral muscles of Longsheng-Feng chickens and AA broiler chickens with different growth rates. Through comprehensive bioinformatics analysis, we identified differentially expressed genes that affect muscle growth and showed that IGF2BP1 is a key participant in chicken muscle development. Subsequently, we employed QRT-PCR, EdU staining, and flow cytometry to further investigate the role of IGF2BP1 in the proliferation and differentiation of chicken myogenic cells. We identified 1143 differentially expressed genes, among which IGF2BP1 is intimately related to the muscle development process and is highly expressed in muscle tissues. Overexpression of IGF2BP1 significantly promotes the proliferation and differentiation of chicken primary myoblasts, while knockdown of IGF2BP1 significantly inhibits these processes. In summary, these results provide valuable preliminary insights into the regulatory roles of IGF2BP1 in chicken growth and development.

## 1. Introduction

Poultry meat, as one of the most important food sources for consumers, undergoes changes in muscle quality that have a significant impact on the meat industry [1]. Muscle development is intrinsically linked to the economic benefits of the poultry industry, and the amount of meat produced is directly dependent on the muscle content [2]. Muscles are primarily classified into three types, skeletal muscle, cardiac muscle, and smooth muscle, among which skeletal muscle is the primary component [3,4]. The development process of skeletal muscle is highly complex, involving an intricate network of interactions among various functional genes [5], regulatory factors [6], and signaling pathways [3,7]. The formation of muscle fibers in the embryonic stage is associated with the proliferation and differentiation of myoblasts. Myoblasts proliferate and differentiate to form myotubes that further mature into muscle fibers [8].The proliferation and differentiation of myogenic cells can to some extent reflect the muscle development process [6]. Therefore, exploring the gene regulatory patterns and interaction networks in skeletal muscle development is essential for revealing the overall mechanisms of muscle development. The precise regulatory network of muscle development has yet to be fully constructed, and its molecular genetic mechanisms continue to require further exploration.

Transcriptomics (RNA-seq) allows for a comprehensive study of transcriptional changes at the whole-organism level, focusing on the transcription and molecular patterns of transcriptional regulation in cells or organ tissues [9]. This technique is widely used to explore the associations between function and phenotype [10,11], and there is a growing body of research on the transcriptomes of various traits in chickens [12,13]. Presently, transcriptomic studies of muscle tissue predominantly focus on the identification of genes regulating the growth and development of muscle tissue [12]. Nevertheless, many studies fall short by not delving deeper into the exploration of these identified key genes, thereby limiting the guidance they offer in comprehending the muscle development process.

IGF2BP1 belongs to the insulin-like growth factor 2 mRNA binding protein family (IGF2BPs/IMPs), serving as a highly conserved post-transcriptional regulatory factor involved in RNA processing, localization, and stability [14,15]. Research suggests that IGF2BP1 plays a pivotal role in embryonic development and tumorigenesis [16,17]. Notably, IGF2BP1 exhibits high expression levels from fertilization to the embryonic period, becoming nearly absent in normal adult organisms [18]. Throughout human embryonic development, IGF2BP1 displays widespread expression in organs but is minimally expressed in the adult prostate, testes, kidneys, and ovaries [19]. Additionally, IGF2BP1 is implicated in the growth and development of animals. The IGF2BP1 gene participates in the co-regulation of key genes during pig embryonic skeletal muscle development through m6A modification [20]. Zhan found that IGF2BP1 was highly expressed in the early stages of goat muscle development [21], while Xu demonstrated that IGF2BP1 promotes the proliferation of goat skeletal muscle satellite cells [22]. In poultry, IGF2BP1 emerges as a key gene regulating the size of Beijing ducks [23], and mutations in the IGF2BP1 gene promoter region, specifically the L1 and L2 types, are significantly associated with multiple growth and body size traits in chickens [24]. Nevertheless, the precise role of IGF2BP1 in the growth and development of chicken skeletal muscle remains unclear.

Arbor Acres (AA) broilers are globally recognized as a fast-growing commercial meat chicken breed, distinguished for their rapid growth and high meat production. In contrast, Longsheng-Feng (LSF) chickens represent a local Chinese breed characterized by traits such as tender meat, robust disease resistance, high adaptability, cold resistance, tolerance to coarse feed, and slower growth. This study analyzed RNA sequencing data from the pectoral muscle tissues of two distinct broiler breeds, identifying IGF2BP1 as a potential regulatory gene that is highly expressed in muscle tissues. Employing both interference and overexpression techniques, the study further investigated the effects of IGF2BP1 on the proliferation and differentiation of chicken myoblasts. This research lays a new foundation for further exploration of the molecular mechanisms underlying chicken muscle development and a detailed analysis of chicken growth.

## 2. Materials and Methods

### 2.1. Animal and Sample Collection

The AA broilers and Longsheng-Feng samples used in this experiment were obtained from the teaching and experimental base of the College of Animal Science and Technology at Guangxi University. The chicken groups were raised until 120 days of age in the same location without restricted feeding. Three female individuals from each group were selected for slaughter, and the same-side breast muscle tissues were collected. Specific information about the samples can be found in Table S1. Additionally, tissues such as heart, liver, spleen, lung, kidney, proventriculus, gizzard, breast muscle, thigh muscle, and abdominal fat tissues were collected for tissue expression profile analysis. E10, E15, 1D, 42D, and 90D pectoral muscle tissues were collected for subsequent temporal expression profiling. All sample tissues were rapidly frozen in liquid nitrogen and transferred to −80 °C for permanent storage until RNA extraction.

### 2.2. Total RNA Extraction and RNA-Seq

Following the manufacturer’s instructions, total RNA was extracted from pectoral muscle tissues using Trizol reagent (Life Technologies, Carlsbad, CA, USA). The purity of the total RNA was assessed through agarose gel electrophoresis. The method for extracting RNA from cells is consistent with that used for tissues, except that grinding is not required. RNA samples from six pectoral muscle tissues that passed quality control were sent to NOVOGENE for library construction and subsequent sequencing.

### 2.3. RNA-Seq Sequencing Data Analysis

After sequencing data were obtained, raw reads were initially filtered to obtain clean reads. FastQC was used for overall quality control of the clean data, including the assessment of error rate, GC content, Q20, and Q30. This ensured that the quality of clean reads met the criteria for subsequent bioinformatics analysis. Subsequently, Hisat2 v2.2.1 was employed to align clean reads with the chicken reference genome. The version of the chicken reference genome used is GRCg6a. Both the genome file and the annotation file were downloaded from Ensembl. HTseq-count v0.6.1p1 was then used to calculate the count for each gene. Finally, differential expression analysis was performed using the DESeq2R v1.34.0 package, comparing AA and LSF. Genes with *p* < 0.05 and |log2FoldChange| ≥ 1 were selected as differentially expressed genes.

### 2.4. Bioinformatics Analysis

Gene Ontology (GO) analysis can predict the potential biological processes and functions that might be affected. Kyoto Encyclopedia of Genes and Genomes (KEGG) analysis was performed to identify the functional molecular pathways possibly involving differentially expressed genes. The clusterProfiler (v4.0.5) R package was utilized for GO and KEGG enrichment analysis of differentially expressed genes. Protein-protein interaction (PPI) analysis was conducted using the STRING database, and visualization was carried out using Cytoscape (v3.10.1) software.

### 2.5. cDNA Synthesis and Real-Time Fluorescent Quantitative PCR (QRT-PCR)

HiScript^®^ III RT SuperMix for qPCR (+gDNA wiper) (Vazyme, Nanjing, China) was utilized for reverse transcription to synthesize cDNA. QRT-PCR was conducted with the following program: the total reaction volume was 20 μL, including 2 μL cDNA, 0.5 μL primers, 10 μL ChamQ Universal SYBR qPCR Master Mix (Vazyme, Nanjing, China), and 7 μL RNase-free water. The QRT-PCR reaction was conducted as follows: 95 °C for 3 min; 95 °C for 10 s, annealing temperature for 30 s for 35 cycles; and finally, melting curve collection at 65 to 95 °C. The accuracy verification of the transcriptome data, the expression of the IGF2BP1 gene, the tissue expression profile of the IGF2BP1 gene, and the expression of proliferation and differentiation marker genes were all validated using QRT-PCR. *β-Actin* was used as the reference gene to normalize gene expression levels. The relative expression levels of the target genes were calculated using the 2^−ΔΔCT^ method. All primers used in this study were synthesized by Sangon Biotech (Sangon, Shanghai, China), and the primer sequences are provided in Table S2.

### 2.6. Isolation and Culture of Chicken Primary Myoblasts (CPMs)

CPMs were isolated from the pectoral muscles of Longsheng-Feng chicken embryos on embryonic day 11 (E11). The recovered CPMs were suspended in growth medium, consisting of Dulbecco’s Modified Eagle Medium (DMEM) (Gibco, Waltham, MA, USA) containing 20% fetal bovine serum (FBS, Gibco, Waltham, MA, USA) and 1% penicillin-streptomycin solution (Solarbio, Beijing, China). The cells were cultured at 37 °C with 5% CO_2_ until reaching 80% confluence. Proliferating cells were collected, or the medium was replaced with differentiation medium containing DMEM, 2% horse serum (HS, Gibco, Waltham, MA, USA), and 1% penicillin-streptomycin solution to induce cell differentiation. Experiments were conducted after 2 days of cultivation in the differentiation medium (DM2).

### 2.7. Plasmid, siRNA, and Transfection

The coding sequence (CDS) fragment of the IGF2BP1 gene was obtained, and an overexpression plasmid was constructed by inserting the CDS into the *XhoI* and *HindIII* restriction enzyme sites of the pEGFP-N1 vector (Promega, WI, USA). siRNA was synthesized by Sangon Biotech (Shanghai, China), and the primer sequences along with interference sequences are provided in Table S2. Lipofectamine 3000 (Invitrogen, Carlsbad, CA, USA) and Lipofectamine RNAiMAX Reagent (Invitrogen, Carlsbad, CA, USA) were used to transfect the plasmid or siRNA into cells with 60–70% confluence in the growth medium. After 24 h of transfection, the medium was replaced with differentiation medium to induce cell differentiation.

### 2.8. 5-Ethynyl-2′-deoxyuridine (EdU) Assay

CPMs were cultured in a 96-well cell plate. After 24 h of transfection, cell proliferation was detected using the Cell-LightTM EdU Apollo In Vitro Kit (RiboBio, Guangzhou, China). Subsequent processing was carried out according to the kit’s instructions, and images were captured using a fluorescence inverted microscope (Zeiss, Germany) in three random fields. Image analysis was then performed using ImageJ software (1.50i) (NIH, Bethesda, MD, USA).

### 2.9. Flow Cytometry Analysis of Cell Cycle

CPMs were cultured in a six-well cell plate, and after 24 h of transfection, the cells were collected. The collected CPM were washed with PBS to remove the supernatant. Subsequently, following the instructions of the Cell Cycle Staining Kit (MultiSciences, Hangzhou, China), further experiments were conducted. Finally, the cells were analyzed using the Attune^®^ NxT Acoustic Focusing Flow Cytometer (ThermoFisher Scientific, Waltham, MA, USA) with the lowest sample injection speed. Each group was subjected to three biological replicates.

### 2.10. Statistical Analysis

All results are presented as the mean ± SEM. Statistical analysis of differences between groups was performed using a two-tailed Student’s *t*-test, and *p* < 0.05 was considered statistically significant; * *p* < 0.05, ** *p* < 0.01. GraphPad Prism 8 (GraphPad Software, San Diego, CA, USA) was employed for data visualization and analysis. All data are presented as the results of three technical and biological replicates.

## 3. Results

### 3.1. Summary of RNA-Seq Data

The transcriptome sequencing results and alignment information are presented in Table 1. After quality control and filtering, we obtained a total of 83.28 G clean reads. The Q20 content exceeded 97%. In addition, we assessed the GC content distribution of clean reads and observed a uniform distribution in the samples, with an average GC content of 54.43%. Further comparison of the clean reads with the reference genome showed a mapping rate exceeding 92%. This indicates that the sequencing data are of high quality and are suitable for subsequent bioinformatics analysis.

### 3.2. RNA-Seq Data and Identification of Differentially Expressed Genes (DEGs)

**Figure 1:**
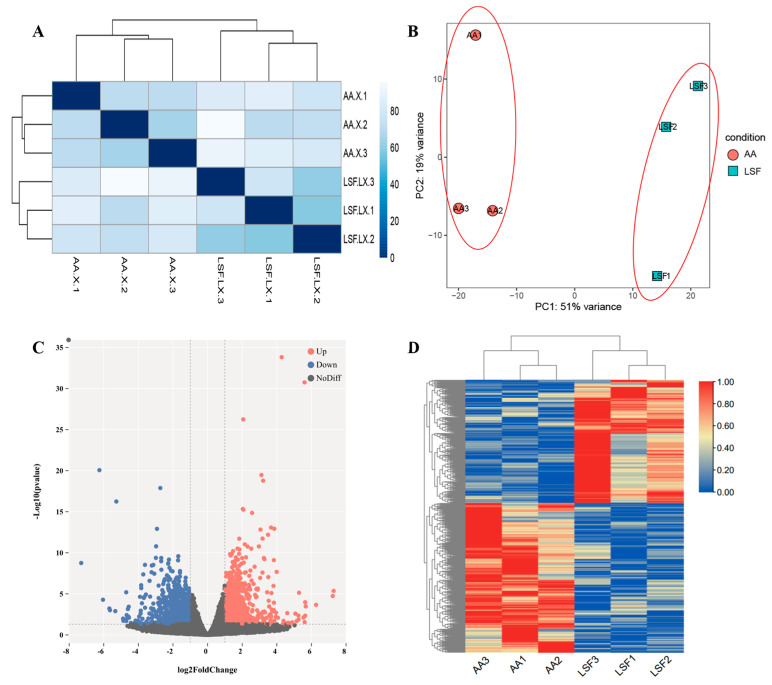
Identification of RNA-Seq Data and Differentially Expressed Genes (DEGs). (**A**) Correlation analysis between samples. (**B**) Principal component analysis. (**C**) Volcano plot depicting the distribution of differentially expressed genes. (**D**) Hierarchical clustering of differentially expressed gene expression.

To explore the expression patterns of genes influencing muscle development in chickens with different growth rates, we utilized transcriptome sequencing technology and the FPKM method for the quantitative analysis of gene expression levels. The correlation coefficient of gene expression levels between samples exceeded 0.8, which indicates that the selected experimental samples can be considered effective biological replicates (Figure 1A). The results of principal component analysis (PCA) in Figure 1B clearly differentiate between the two treatment groups, with the AA group (Group 1) and LSF group (Group 2) clustering together. No crossover between different breeds was observed, indicating appropriate biological replicates and significant inter-group differences, and thereby reinforcing the reliability of the samples. Differential expression analysis detected 1143 DEGs in the pectoral muscle tissue, including 543 downregulated genes and 600 upregulated genes (Figure 1C and Table S3) The hierarchical clustering heatmap demonstrates that AA broilers and Longsheng-Feng chickens cluster separately, exhibiting relatively consistent expression patterns of differentially expressed genes within each group (Figure 1D).

### 3.3. GO and KEGG Pathway Analysis of DEGs

**Figure 2:**
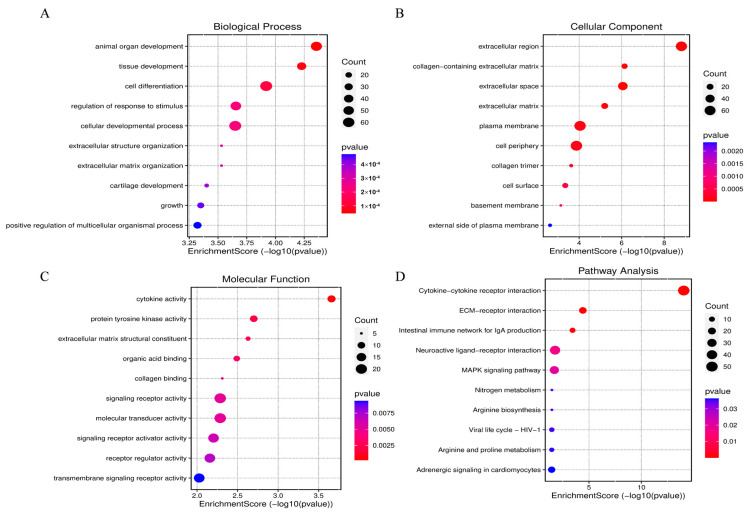
Bioinformatics Analysis of Differentially Expressed Genes (DEGs). (**A**) Enriched terms in biological processes for DEGs. (**B**) Enriched terms in cellular components for DEGs. (**C**) Enriched terms in molecular functions for DEGs. (**D**) KEGG enrichment analysis of DEGs.

To elucidate the functions of DEGs, GO enrichment analysis was utilized to annotate and explore their distribution. A total of 1143 DEGs were found to be enriched in 228 different GO terms (Table S4). The top 10 significantly enriched GO terms are displayed in Figure 2A–C. The molecular functions of the genes primarily involved animal organ development, tissue development, and cell differentiation (Figure 2A); cellular components related to these genes include the extracellular region, collagen-containing extracellular matrix, and extracellular space (Figure 2B), and biological processes include cytokine activity, protein tyrosine kinase activity, and extracellular matrix structural constituent (Figure 2C). Additionally, the main enriched KEGG pathways in differentially expressed genes pertain to the regulation of skeletal muscle development, including the MAPK signaling pathway, cytokine-cytokine receptor interaction, adrenergic signaling in cardiomyocytes, and regulation of amino acid biosynthesis (Figure 2D and Table S5).

### 3.4. PPI Interaction Analysis and Selection of Target Genes

**Figure 3:**
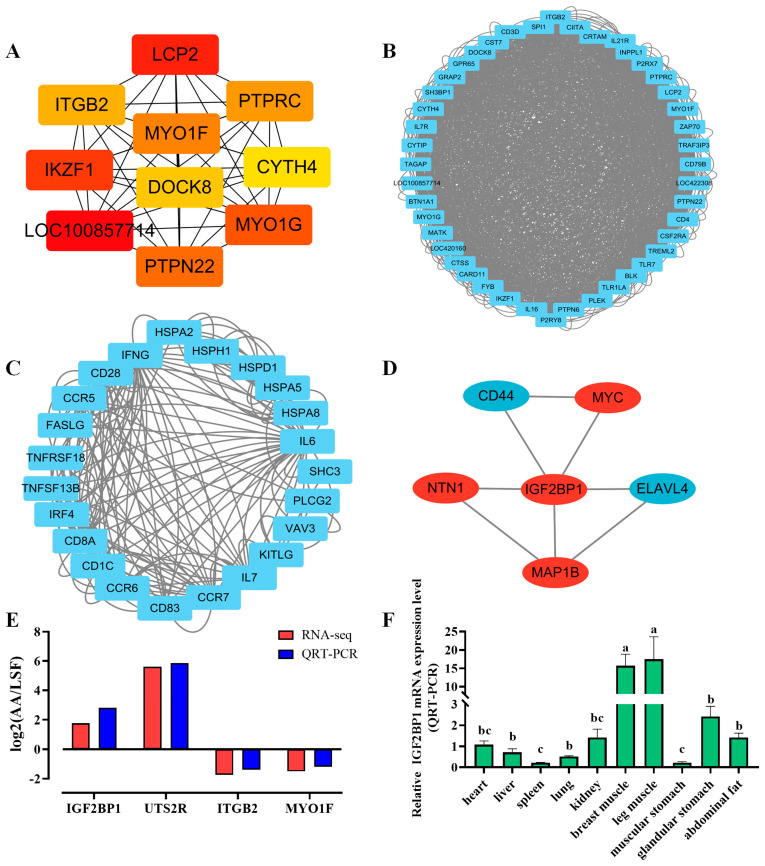
Analysis of protein-protein interactions of differentially expressed genes and screening of candidate genes. (**A**) Top 10 hub genes identified by Cytoscape CytoHubba. (**B**,**C**) Top 3 protein-protein interaction clusters. (**D**) Protein-protein interaction cluster of IGF2BP1. (**E**) Validation of RNA-seq data by QRT-PCR. (**F**) Expression of IGF2BP1 in 10 different chicken tissues measured by QRT-PCR (*n* = 6). Significance between groups is expressed using alphabetical notation, where the same letter indicates that the differences are not significant.

To further reveal the interaction relationships among differentially expressed genes and identify core candidate genes, protein-protein interaction (PPI) analysis was conducted. The results showed a total of 7458 interaction relationships, and the top three interaction modules were identified (Figure 3A–C). Additionally, using the sequencing results and references related to muscle development, we analyzed and selected differentially expressed genes associated with important GO terms, KEGG pathways, and PPIs. These genes are IGF2BP1, NTN1, CD44, MYC, ELAVL4, MAP1, BLCP2, MYO1F, PTPRC, and ITGB2 (Figure 3D). Subsequently, four differentially expressed genes were randomly selected to validate the accuracy of the sequencing data, and the results confirmed that the expression trends of these genes were consistent between QRT-PCR and RNA-seq (Figure 3E). Following that, IGF2BP1 was designated as the main research target based on previous studies by the collaborative research group. The expression of the IGF2BP1 gene was then detected in 10 different chicken tissues. Based on real-time quantitative PCR results, it was found that the expression levels of the IGF2BP1 gene in the selected 10 tissues were significantly higher in pectoral muscle and leg muscle compared with other tissues (Figure 3F). In the analysis of temporal expression in pectoral muscle tissue, the expression level of the IGF2BP1 gene was observed to gradually increase over time during the embryonic period (Supplementary Figure S1A). Finally, we examined the temporal expression of the IGF2BP1 gene during the proliferation and differentiation phases at the cellular level and observed that its expression level initially increased and then decreased during the differentiation period, with a peak on the third day. The IGF2BP1 gene is speculated to be involved in the myoblast differentiation process (Supplementary Figure S1B).

### 3.5. Interfering with IGF2BP1 Inhibits Proliferation and Differentiation of CPM

**Figure 4:**
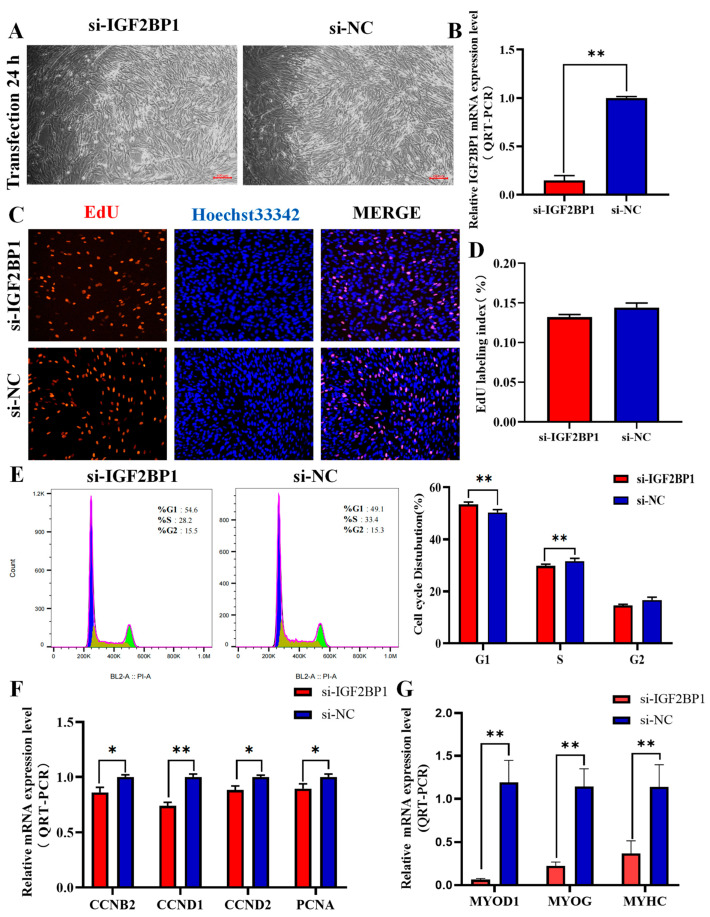
Effects of IGF2BP1 Interference on the Proliferation and Differentiation of Chicken Skeletal Muscle Cells. (**A**) Light microscopy images of chicken skeletal muscle cells before and after transfection, 100×. (**B**) Relative mRNA expression level of IGF2BP1 after interference. (**C**) EdU fluorescence staining, 100×. (**D**) EdU-positive cell rate. (**E**) Flow cytometry analysis of the cell cycle after IGF2BP1 interference. (**F**) QRT-PCR detection of the mRNA expression levels of proliferation marker genes in chicken skeletal muscle cells transfected with si-IGF2BP1 or si-NC. (**G**) QRT-PCR detection of the mRNA expression levels of differentiation marker genes in chicken skeletal muscle cells transfected with si-IGF2BP1 or si-NC. si-NC represents negative control. * *p* < 0.05, ** *p* < 0.01.

To further explore the role of IGF2BP1 in the proliferation of chicken primary myoblasts (CPMs), si-IGF2BP1 and si-NC were transfected into CPM, and QRT-PCR was utilized to measure the relative expression of IGF2BP1 at the mRNA level. The results in Figure 4A,B show that, compared with the control group, the designed si-IGF2BP1 fragment significantly reduced the expression of the IGF2BP1 gene (*p* < 0.01); Therefore, this fragment is suitable for further experiments. Subsequently, si-IGF2BP1 was transfected and the expression of genes regulating cell cycle-related factors was assessed, including Cyclin B2 (CCNB2), Cyclin D1 (CCND1), Cyclin D2 (CCND2), and Proliferating Cell Nuclear Antigen (PCNA). The results in Figure 4F demonstrate that, compared with si-NC, IGF2BP1 interference led to a significant downregulation of CCNB2, CCND1, CCND2, and PCNA expression (*p* < 0.05). EdU incorporation assay results indicate that the proportion of EdU-positive cells was reduced with si-IGF2BP1 compared with si-NC (*p* > 0.05, Figure 4C,D). Additionally, cell cycle analysis (Figure 4E) revealed that IGF2BP1 interference increased the proportion of G1-phase cells and reduced the proportion of S-phase cells compared to the control group, suggesting inhibition of cell division. This proportion is significantly higher than the control group (*p* < 0.01). After the induction of differentiation in transfected cells, imaging revealed that, in comparison to the control group, myoblast differentiation in cells transfected with si-IGF2BP1 was significantly inhibited (Supplementary Figure S2A). Then, QRT-PCR was used to measure the mRNA expression levels of marker genes related to cell differentiation, including Myogenic Differentiation Factor 1 (MYOD1), Myogenin (MYOG), and Myosin Heavy Chain (MYHC). The results in Figure 4G demonstrate that interfering with IGF2BP1 significantly reduced the mRNA expression levels of MYOD1, MYOG, and MYHC (*p* < 0.01). These results indicate that interfering with IGF2BP1 can inhibit the proliferation and differentiation of CPMs.

### 3.6. Overexpression of IGF2BP1 Promotes Proliferation and Differentiation of CPM

**Figure 5:**
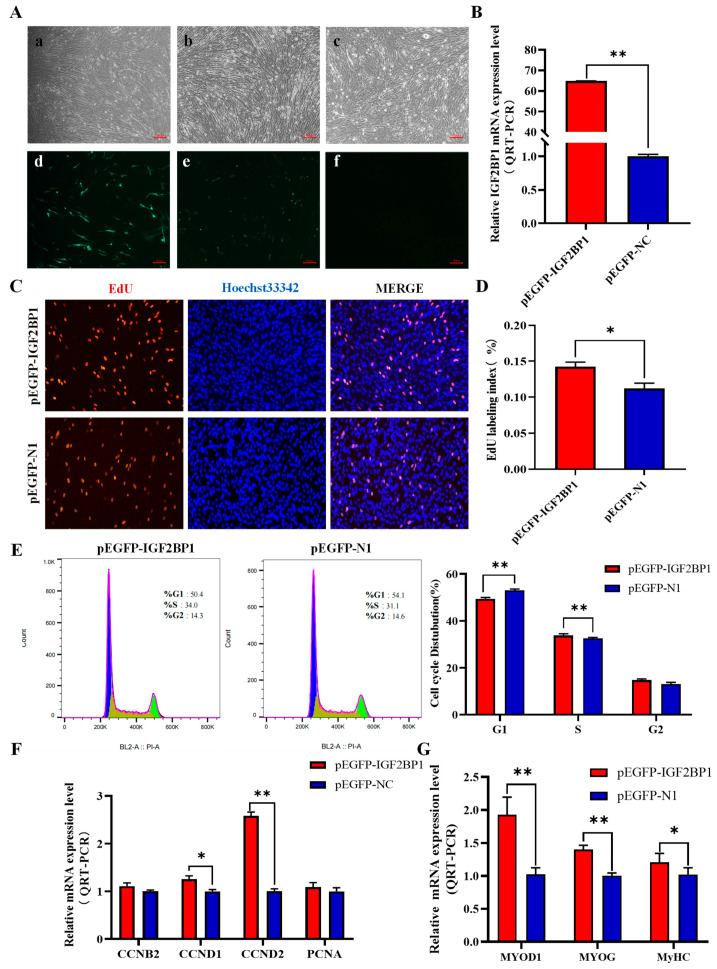
Effects of IGF2BP1 Overexpression on the Proliferation and Differentiation of Chicken Skeletal Muscle Cells. (**A**) 100×, images of chicken skeletal muscle cells 48 h after transfection with pEGFP-IGF2B1, (**a**,**d**) pEGFP-N1, (**b**,**e**) pEGFP-IGF2BP1, and (**c**,**f**) negative control. (**B**) Relative mRNA expression level of IGF2BP1 after overexpression. (**C**) EdU fluorescence staining, 100×. (**D**) EdU-positive cell rate. (**E**) Flow cytometry analysis of the cell cycle after IGF2BP1 overexpression. (**F**) QRT-PCR detection of the mRNA expression levels of proliferation marker genes in chicken skeletal muscle cells transfected with pEGFP-IGF2B1 or pEGFP-N1. (**G**) QRT-PCR detection of the mRNA expression levels of differentiation marker genes in chicken skeletal muscle cells transfected with pEGFP-IGF2B1 or pEGFP-N1. pEGFP-N1 represents negative control. * *p* < 0.05, ** *p* < 0.01.

To further elucidate the function of IGF2BP1 in chicken muscle, the pEGFP-IGF2BP1 vector was successfully constructed and transfected into CPM alongside pEGFP-N1 (Figure 5A). The results demonstrated a highly significant efficiency of IGF2BP1 overexpression (*p* < 0.01), thereby rendering it suitable for subsequent experiments (Figure 5B). After transfection, changes in the expression of proliferation-related marker genes were detected using QRT-PCR. The mRNA levels of proliferation marker genes, including CCND1 and CCND2, significantly increased after IGF2BP1 overexpression (Figure 5F). EdU incorporation assay results indicated a significant increase in the proportion of EdU-positive cells with IGF2BP1 overexpression compared with the control group (*p* < 0.05, Figure 5C,D). Cell cycle analysis results revealed a decrease in the proportion of G1-phase cells and an increase in the proportion of S-phase cells after IGF2BP1 overexpression, suggesting that the overexpression of IGF2BP1 promotes cell division (Figure 5E). Following transfection, we observed the cells induced to differentiate and noted that they displayed a more pronounced differentiation state relative to the control group (Supplementary Figure S2B). Subsequently, the expression of marker genes related to cell differentiation was examined after IGF2BP1 overexpression. The results in Figure 5G demonstrate that the overexpression of IGF2BP1 significantly promotes the mRNA expression of MYOD1, MYOG, and MYHC (*p* < 0.01). The above results suggest that the overexpression of IGF2BP1 can promote the proliferation and differentiation of CPMs. Based on the findings of this study, it was discovered that IGF2BP1 can promote the proliferation and differentiation of chicken myoblasts.

## 4. Discussion

Recently, RNA-seq has gained extensive application in poultry muscle research, serving as a crucial tool for elucidating muscle development, disease mechanisms, and gene regulation [25]. AA broilers and Longshen-Feng chickens exhibit significant differences in meat production, growth rate, and disease resistance [26,27]. This study involved collecting transcriptome sequencing data from the pectoral muscle tissues of AA broilers and LSF chickens of the same age, revealing 543 downregulated and 600 upregulated differential genes between the two breeds.

Functional enrichment analysis was conducted on the identified differentially expressed genes. GO enrichment analysis showed that these genes are primarily involved in biological processes related to the extracellular matrix, including the collagen-containing extracellular matrix. The extracellular matrix plays a pivotal role in muscle development and repair, regulating growth, differentiation, the maintenance of the structure and morphology of muscle cells, and interactions with surrounding tissues. The extracellular matrix in muscle tissue is composed of molecules such as collagen, fibronectin, and proteoglycans, which provide support and structure to muscle cells and participate in various biological processes in muscle [28]. Cell adhesion, migration, proliferation, and differentiation are closely associated with the extracellular matrix [8,29]. The migration, proliferation, and differentiation of myoblasts is fundamental to myogenesis, which is essential for skeletal muscle hypertrophy and regeneration [30,31]. KEGG enrichment analysis demonstrated that differentially expressed genes are mainly involved in cytokine-cytokine receptor interaction, adrenergic signaling in cardiomyocytes, and the MAPK signaling pathway. In muscle development, some cytokines and their receptors are involved in processes such as the proliferation, differentiation, and repair of muscle cells. For example, increased expression of the IL-6 gene in muscle cells can inhibit the proliferation, differentiation, and migration of myoblasts and skeletal muscle satellite cells [32]. The MAPK signaling pathway is crucial for muscle development, [33],and in chickens, it is mediated by PDLIM5, regulating the development of skeletal muscle satellite cells [34].

Research shows a high correlation between IGF2BP1 and the early embryo development as well as the growth traits of animals. Wu discovered that the IGF2BP1 gene is essential for liver growth during zebrafish embryonic development [35]. Yaniv, in studies of embryos of African clawed frogs, zebrafish, and mice, observed the expression of this gene in various developmental cell types [36]. The IGF2BP1 gene can co-regulate key genes in porcine embryonic skeletal muscle development through m6A [20]. In poultry, Zhou discovered a long-distance mutation near the IGF2BP1 gene in Beijing ducks, which led to a 15% increase in body size and a 6% improvement in feed efficiency [23].Additionally, Wang found associations between the IGF2BP1 gene and traits such as body weight and body size in chickens [24]. Through tissue expression profile analysis, this study identified significantly higher expression levels of the IGF2BP1 gene in breast muscle and leg muscle compared with other tissues, indicating a potential role for the IGF2BP1 gene in muscle.

Gene overexpression and interference techniques are important tools for studying gene function [37,38]. Research has shown that the level of active gene expression reflects the progress of muscle development. Examples of such genes include CCNB2, CCND1, CCND2, and PCNA [39]. These genes are associated with the exit of muscle cells from the cell cycle, and the expression of muscle-specific genes, as well as the inhibition of the expression of other cell- or tissue-specific genes. Therefore, in this study, through the analysis of the overexpression and interference of IGF2BP1 in relevant cell experiments, it was found that interfering with IGF2BP1 led to the significant downregulation of key proliferation genes such as CCNB2, CCND1, CCND2, and PCNA in muscle cells, while overexpressing IGF2BP1 promoted the upregulation of CCND1 and CCND2. The flow cytometry results showed that interference with IGF2BP1 reduced the proportion of cells in the S phase while increasing the proportion in the G1 phase. Conversely, the overexpression of IGF2BP1 increased the number of muscle cells entering the S phase and decreased the number entering the G0/G1 phase. Additionally, the EdU results demonstrated that the overexpression of IGF2BP1 significantly promoted the vitality and proliferation of muscle cells, while interference with IGF2BP1 showed the opposite trend. Therefore, our study demonstrates that IGF2BP1 is crucial for the proliferation of chicken muscle cells.

Skeletal muscle differentiation is a complex, multi-step process involving myoblasts fusing to form muscle fibers. Muscle fibers are tubular, multinucleated structures that contain the contraction machinery necessary for muscle contraction [40]. To determine if IGF2BP1 affects the differentiation of myoblasts, we analyzed its temporal expression in chicken pectoral muscle tissues across various developmental stages, observing a progressive increase in expression during embryonic days E10 and E15. The period from embryonic day E12 to E16 is recognized as a critical phase for the proliferation and differentiation of myoblasts into muscle fibers [41]. This expression profile indicates that IGF2BP1 is likely to be involved in the regulatory mechanisms critical to the growth and differentiation of myoblasts into mature muscle fibers. To investigate the specific role of IGF2BP1 in the differentiation of chicken myoblasts, we examined CPMs that were induced to differentiate following transfection with both interfering and overexpressing constructs. The findings indicated that the overexpression of IGF2BP1 enhanced the differentiation process, as evidenced by a marked increase in the number of myotubes. By examining the expression levels of the differentiation-related marker genes MYOD1, MYOG, and MYHC in CPMs, we investigated the impact of IGF2BP1 on CPM differentiation. The results demonstrated a significant increase in the expression of differentiation marker genes following the overexpression of IGF2BP1, while the opposite effects were noted following the interference of IGF2BP1. These findings suggest an association between IGF2BP1 and the differentiation of myoblasts. In summary, IGF2BP1 has been shown to promote the differentiation of chicken primary myoblasts.

## 5. Conclusions

In conclusion, transcriptome sequencing has identified 10 candidate genes potentially regulating muscle development. We selected one gene, IGF2BP1, noted for its high expression specifically in chicken muscle tissues. Interference with IGF2BP1 can suppress the proliferation and differentiation of CPMs, while its overexpression may enhance these processes. These results indicate that IGF2BP1 plays a crucial role in the growth and development of chicken muscles.

## Figures and Tables

**Table 1:** Summary of sequencing reads mapping to the reference genome and quality parameters.

Sample Name	Clean Reads	Clean Base(G)	Q20(%)	Content (%)	Mapping Rate (%)
AA1	82,372,256	12.36	97.61	53.67	93.37
AA2	76,265,758	11.44	97.74	56.45	93.31
AA3	90,003,894	13.5	97.62	56.85	92.87
LSF1	103,112,308	15.47	97.34	53.12	92.33
LSF2	107,514,536	15.67	97.18	54.05	92.37
LSF3	98,934,380	14.84	97.19	52.46	92.29

## Data Availability

The original contributions presented in the study are included in the article/Supplementary Materials, further inquiries can be directed to the corresponding author.

## Supplementary Materials

The following supporting information can be downloaded at https://www.mdpi.com/article/10.3390/ani14142024/s1: Figure S1: Expression of IGF2BP1 in chicken pectoral muscle tissue and chicken primary myoblasts; Figure S2: Light microscopy images of CPMS differentiation after transfection interference and overexpression; Table S1: RNA-seq Sampling Information; Table S2: Interference and primer sequence information; Table S3: Screening for AA vs. LSF differentially expressed genes; Table S4: GO terms enriched by DEGs; Table S5: KEGG pathway enriched by DEGs.

## References

[1] Ren L., Liu A., Wang Q., Wang H., Dong D., Liu L. (2021). Transcriptome analysis of embryonic muscle development in Chengkou Mountain Chicken. BMC Genom..

[2] Hayes V.E., Hikida R.S. (1976). Naturally-occurring degeneration in chick muscle development: Ultrastructure of the *M. complexus*. J. Anat..

[3] Khabyuk J., Prols F., Draga M., Scaal M. (2022). Development of ribs and intercostal muscles in the chicken embryo. J. Anat..

[4] Rahmati M., Mccarthy J.J., Malakoutinia F. (2022). Myonuclear permanence in skeletal muscle memory: A systematic review and meta-analysis of human and animal studies. J. Cachexia Sarcopenia Muscle.

[5] Lin S., Xian M., Ren T., Mo G., Zhang L., Zhang X. (2022). Mining of chicken muscle growth genes and the function of important candidate gene RPL3L in muscle development. Front. Physiol..

[6] Ren P., Chen M., Li J., Lin Z., Yang C., Yu C., Zhang D., Liu Y. (2023). MYH1F promotes the proliferation and differentiation of chicken skeletal muscle satellite cells into myotubes. Anim. Biotechnol..

[7] Zi J., Xu J., Luo J., Yang X., Zhen Z., Li X., Hu D., Guo Y., Guo H., Ding X. (2022). PFN1 Inhibits Myogenesis of Bovine Myoblast Cells via Cdc42-PAK/JNK. Cells.

[8] Ferreira M.M., Dewi R.E., Heilshorn S.C. (2015). Microfluidic analysis of extracellular matrix-bFGF crosstalk on primary human myoblast chemoproliferation, chemokinesis, and chemotaxis. Integr. Biol..

[9] Withanage M., Liang H., Zeng E. (2022). RNA-Seq Experiment and Data Analysis. Methods Mol. Biol..

[10] Peng L.Y., Cui Z.Q., Wu Z.M., Fu B.D., Yi P.F., Shen H.Q. (2019). RNA-seq profiles of chicken type II pneumocyte in response to Escherichia coli infection. PLoS ONE.

[11] Yu B., Liu J., Cai Z., Wang H., Feng X., Zhang T., Ma R., Gu Y., Zhang J. (2023). RNA N (6)-methyladenosine profiling reveals differentially methylated genes associated with intramuscular fat metabolism during breast muscle development in chicken. Poult. Sci..

[12] Cao C., Cai Y., Li Y., Li T., Zhang J., Hu Z., Zhang J. (2023). Characterization and comparative transcriptomic analysis of skeletal muscle in female Pekin duck and Hanzhong Ma duck during different growth stages using RNA-seq. Poult. Sci..

[13] Xing S., Liu R., Zhao G., Liu L., Groenen M., Madsen O., Zheng M., Yang X., Crooijmans R., Wen J. (2020). RNA-Seq Analysis Reveals Hub Genes Involved in Chicken Intramuscular Fat and Abdominal Fat Deposition During Development. Front. Genet..

[14] Chennathukuzhi V., Stein J.M., Abel T., Donlon S., Yang S., Miller J.P., Allman D.M., Simmons R.A., Hecht N.B. (2003). Mice deficient for testis-brain RNA-binding protein exhibit a coordinate loss of TRAX, reduced fertility, altered gene expression in the brain, and behavioral changes. Mol. Cell. Biol..

[15] Ruggiu M., Speed R., Taggart M., Mckay S.J., Kilanowski F., Saunders P., Dorin J., Cooke H.J. (1997). The mouse Dazla gene encodes a cytoplasmic protein essential for gametogenesis. Nature.

[16] Elcheva I.A., Gowda C.P., Bogush D., Gornostaeva S., Fakhardo A., Sheth N., Kokolus K.M., Sharma A., Dovat S., Uzun Y. (2023). IGF2BP family of RNA-binding proteins regulate innate and adaptive immune responses in cancer cells and tumor microenvironment. Front. Immunol..

[17] Huang X., Zhang H., Guo X., Zhu Z., Cai H., Kong X. (2018). Insulin-like growth factor 2 mRNA-binding protein 1 (IGF2BP1) in cancer. J. Hematol. Oncol..

[18] Bell J.L., Wachter K., Muhleck B., Pazaitis N., Kohn M., Lederer M., Huttelmaier S. (2013). Insulin-like growth factor 2 mRNA-binding proteins (IGF2BPs): Post-transcriptional drivers of cancer progression?. Cell. Mol. Life Sci..

[19] Hammer N.A., Hansen T., Byskov A.G., Rajpert-De M.E., Grondahl M.L., Bredkjaer H.E., Wewer U.M., Christiansen J., Nielsen F.C. (2005). Expression of IGF-II mRNA-binding proteins (IMPs) in gonads and testicular cancer. Reproduction.

[20] Zhang D., Wu S., Zhang X., Ren S., Tang Z., Gao F. (2022). Coordinated transcriptional and post-transcriptional epigenetic regulation during skeletal muscle development and growth in pigs. J. Anim. Sci. Biotechnol..

[21] Zhan S., Zhao W., Song T., Dong Y., Guo J., Cao J., Zhong T., Wang L., Li L., Zhang H. (2018). Dynamic transcriptomic analysis in hircine longissimus dorsi muscle from fetal to neonatal development stages. Funct. Integr. Genom..

[22] Xu X., Leng J., Zhang X., Capellini T.D., Chen Y., Yang L., Chen Z., Zheng S., Zhang X., Zhan S. (2021). Identification of IGF2BP1-related lncRNA-miRNA-mRNA network in goat skeletal muscle satellite cells. Anim. Sci. J..

[23] Zhou Z., Li M., Cheng H., Fan W., Yuan Z., Gao Q., Xu Y., Guo Z., Zhang Y., Hu J. (2018). Author Correction: An intercross population study reveals genes associated with body size and plumage color in ducks. Nat. Commun..

[24] Wang K., Hu H., Tian Y., Li J., Scheben A., Zhang C., Li Y., Wu J., Yang L., Fan X. (2021). The Chicken Pan-Genome Reveals Gene Content Variation and a Promoter Region Deletion in IGF2BP1 Affecting Body Size. Mol. Biol. Evol..

[25] Chen J., Zhang S., Chen G., Deng X., Zhang D., Wen H., Yin Y., Lin Z., Zhang X., Luo W. (2022). Transcriptome Sequencing Reveals Pathways Related to Proliferation and Differentiation of Shitou Goose Myoblasts. Animals.

[26] Ren T., Li Z., Zhou Y., Liu X., Han R., Wang Y., Yan F., Sun G., Li H., Kang X. (2018). Sequencing and characterization of lncRNAs in the breast muscle of Gushi and Arbor Acres chickens. Genome.

[27] Sarsenbek A., Wang T., Zhao J.K., Jiang W. (2013). Comparison of carcass yields and meat quality between Baicheng-You chickens and Arbor Acres broilers. Poult. Sci..

[28] Ahmad K., Lee E.J., Moon J.S., Park S.Y., Choi I. (2018). Multifaceted Interweaving Between Extracellular Matrix, Insulin Resistance, and Skeletal Muscle. Cells.

[29] Ahmed M., Ffrench-Constant C. (2016). Extracellular Matrix Regulation of Stem Cell Behavior. Curr. Stem Cell Rep..

[30] Bentzinger C.F., Wang Y.X., Rudnicki M.A. (2012). Building muscle: Molecular regulation of myogenesis. Cold Spring Harbor Perspect. Biol..

[31] Braun T., Gautel M. (2011). Transcriptional mechanisms regulating skeletal muscle differentiation, growth and homeostasis. Nat. Rev. Mol. Cell Biol..

[32] Saclier M., Yacoub-Youssef H., Mackey A.L., Arnold L., Ardjoune H., Magnan M., Sailhan F., Chelly J., Pavlath G.K., Mounier R. (2013). Differentially activated macrophages orchestrate myogenic precursor cell fate during human skeletal muscle regeneration. Stem. Cells.

[33] Lehka L., Redowicz M.J. (2020). Mechanisms regulating myoblast fusion: A multilevel interplay. Semin. Cell Dev. Biol..

[34] He H., Yin H., Yu X., Zhang Y., Ma M., Li D., Zhu Q. (2021). PDLIM5 Affects Chicken Skeletal Muscle Satellite Cell Proliferation and Differentiation via the p38-MAPK Pathway. Animals.

[35] Wu J., Lu C., Ge S., Mei J., Li X., Guo W. (2020). Igf2bp1 is required for hepatic outgrowth during early liver development in zebrafish. Gene.

[36] Yaniv K., Fainsod A., Kalcheim C., Yisraeli J.K. (2003). The RNA-binding protein Vg1 RBP is required for cell migration during early neural development. Development.

[37] Hannon G.J. (2002). RNA interference. Nature.

[38] Chen B., Zhang Y., Niu Y., Wang Y., Liu Y., Ji H., Han R., Tian Y., Liu X., Kang X. (2023). RRM2 promotes the proliferation of chicken myoblasts, inhibits their differentiation and muscle regeneration. Poult. Sci..

[39] Chen B., Wang Y., Hou D., Zhang Y., Zhang B., Niu Y., Ji H., Tian Y., Liu X., Kang X. (2023). Transcriptome-Based Identification of the Muscle Tissue-Specific Expression Gene CKM and Its Regulation of Proliferation, Apoptosis and Differentiation in Chicken Primary Myoblasts. Animals.

[40] Daczewska M. (2020). Differentiation of skeletal muscles. Semin. Cell Dev. Biol..

[41] O’Neill M.C. (1987). Growth and differentiation during myogenesis in the chick embryo. Dev. Biol..

