# Supplementary material for: High Muscle Expression of IGF2BP1 Gene Promotes Proliferation and Differentiation of Chicken Primary Myoblasts: Results of Transcriptome Analysis
Source: Animals (Basel). 2024 Jul 9;14(14):2024. doi: 10.3390/ani14142024 (PMC11274093; doi:10.3390/ani14142024)
Supplement: Supplementary file 1 [file animals-14-02024-s001.zip › Supplementary Figure.pdf]

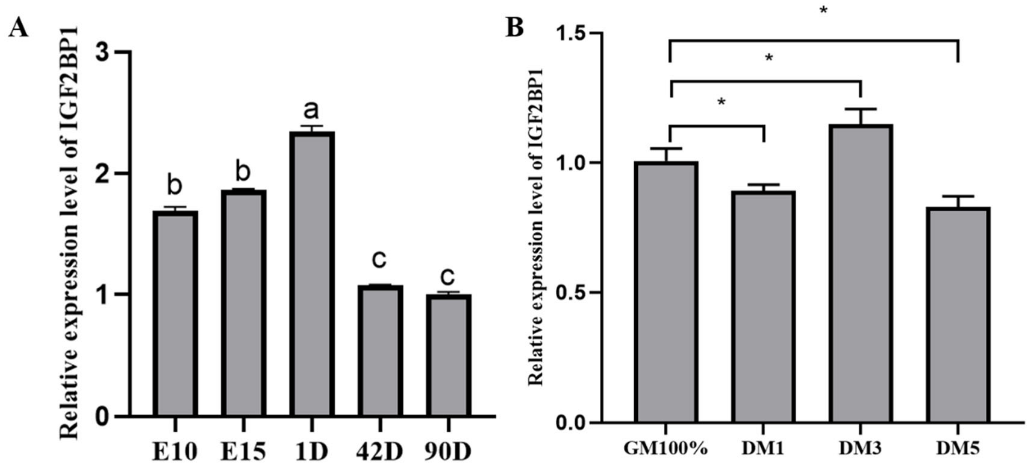

Supplementary Figure S1. Expression of igf2bp1 in chicken pectoral muscle tissue and chicken primary myoblasts. (A) Temporal expression profile of IGF2BP1 gene in pectoral muscle tissues at different times. (B) Temporal expression profile of IGF2BP1 gene in differentiating adult muscle cells.

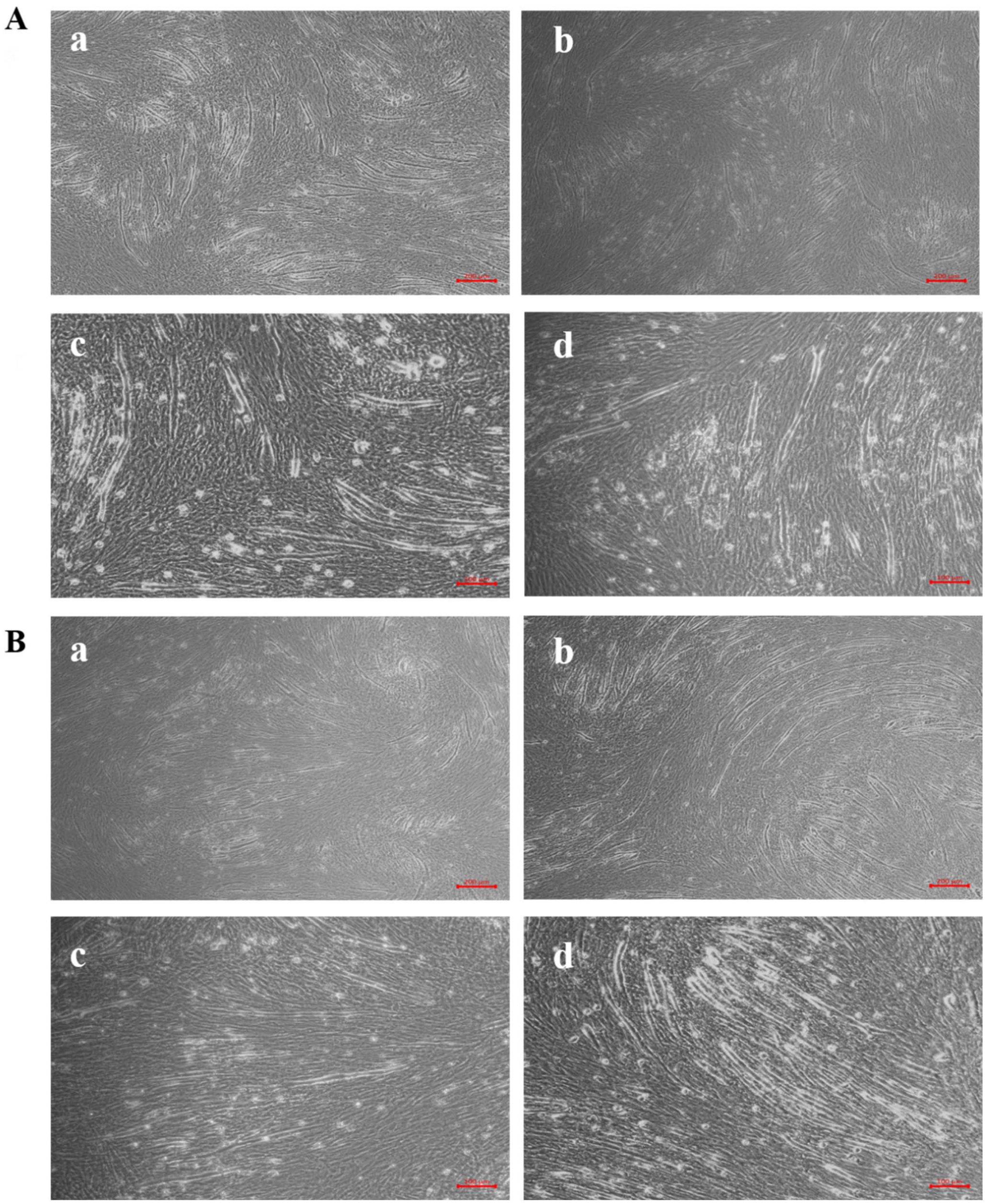

Supplementary Figure S2. Light microscopy images of CPMS differentiation after transfection interference and overexpression. **(**A) Differentiation of adult chicken myoblasts 2 days post-transfection interference, with panels a and c showing si-NC and panels b and d showing si-IGF2BP1. (B) Differentiation of adult chicken myoblasts 2 days post-overexpression, with panels a and c displaying pEGFP-N1 and panels b and d showing pEGFP-IGF2BP1.
